# Limitations to the Use of Species-Distribution Models for Environmental-Impact Assessments in the Amazon

**DOI:** 10.1371/journal.pone.0146543

**Published:** 2016-01-19

**Authors:** Lorena Ribeiro de A. Carneiro, Albertina P. Lima, Ricardo B. Machado, William E. Magnusson

**Affiliations:** 1 Programa de Pós-Graduação em Ecologia, Instituto Nacional de Pesquisas da Amazônia, Manaus, Amazonas, Brazil; 2 Departamento de Zoologia, Universidade de Brasília, Brasília, Distrito Federal, Brazil; Field Museum of Natural History, UNITED STATES

## Abstract

Species-distribution models (SDM) are tools with potential to inform environmental-impact studies (EIA). However, they are not always appropriate and may result in improper and expensive mitigation and compensation if their limitations are not understood by decision makers. Here, we examine the use of SDM for frogs that were used in impact assessment using data obtained from the EIA of a hydroelectric project located in the Amazon Basin in Brazil. The results show that lack of knowledge of species distributions limits the appropriate use of SDM in the Amazon region for most target species. Because most of these targets are newly described and their distributions poorly known, data about their distributions are insufficient to be effectively used in SDM. Surveys that are mandatory for the EIA are often conducted only near the area under assessment, and so models must extrapolate well beyond the sampled area to inform decisions made at much larger spatial scales, such as defining areas to be used to offset the negative effects of the projects. Using distributions of better-known species in simulations, we show that geographical-extrapolations based on limited information of species ranges often lead to spurious results. We conclude that the use of SDM as evidence to support project-licensing decisions in the Amazon requires much greater area sampling for impact studies, or, alternatively, integrated and comparative survey strategies, to improve biodiversity sampling. When more detailed distribution information is unavailable, SDM will produce results that generate uncertain and untestable decisions regarding impact assessment. In many cases, SDM is unlikely to be better than the use of expert opinion.

## Introduction

Species-distribution models (SDM) can be useful in conservation planning [1–3], and they may be used to guide decisions regarding environmental-impact analysis and licensing in Brazil [4]. Environmental licensing is a mandatory procedure, often considered an important instrument for environmental control, and is enforced prior to the installation of facilities or activities that may cause impact [5]. Thus, environmental-impact studies are used by government agencies to assess the environmental liability of the project and make decisions concerning the actions required to mitigate environmental damage.

In many Latin American countries, in addition to mitigation measures to minimize negative effects, the licensing process may also provide compensation for the loss of natural resources due to temporary or permanent use [6]. These environmental offsets are usually the creation or consolidation of protected areas with environmental or biodiversity similarities to the impacted region. Latin-American countries are increasingly investing in economic strategies for expansion of the energy sectors [5] and the Amazon region of Brazil has the largest remaining hydroelectric potential [7–8]. However, the impacts of these programs on local ecosystems and biodiversity are likely to lead to irreversible and global ecological and climatic imbalances [7–10].

SDM has been suggested to be used by licensing agencies as a tool to reduce subjectivity in the environmental-impact analyses, and for mitigation and compensation measures [4]. It is assumed that SDM will indicate where the most environmentally appropriate areas are that will mitigate or offset the damage caused by the activity under consideration.

Limitations to the use of SDM have been suggested, such as when the data available are insufficient to inform the models as to true species distributions [11–13], or when predictions based on extrapolations may not be robust [14–15]. However, these questions have been theoretical and, to date, no study has examined the use of SDM and the decisions that followed with respect to environmental licensing. Here, we use data on frogs collected during the EIA of a hydroelectric plant built in the Amazon basin, to examine whether the data used was adequate to calibrate the SDM at the scales of common mitigation and compensation decisions.

Amphibians are frequently used as biological indicators of human disturbance in terrestrial and aquatic ecosystems, because they are strongly impacted by many human modifications and frequently occur in the riparian areas most affected by the installation of hydroelectric projects. While we use amphibians in this example, our results will apply to any other species used in environmental-impact studies in tropical regions in which true species distributions are poorly known and poorly sampled.

### Target-species and the scales of decisions

Because of time and financial constraints involved in implementing mitigation actions, conservation strategies should target the most impacted species, which we will refer to as target-species [16]. Some criteria to select targets in environmental-impact studies are defined in Brazilian legislation [17–18], but some of them may be inappropriate for use in the Amazon region. For example, the use of conservation status of a species based on IUCN or a national list of threatened species [17–18]. These criteria depend on the spatial scale of the analysis and may be uninformative for poorly known groups of Amazonian fauna, such as frogs that are often classified as “data deficient.” Thus, target species must first be identified, based on probable impact and biological complementarity, to determine data availability for those species that will be used to calibrate the model on the scales at which decisions need to be made.

Licensing agencies may require local to national-scale assessment, depending on the legal and environmental contexts and probable extent of damage. Spatial analysis of impact mitigation requires local-scale models to guide the location of new surveys or of faunal relocation, and at the regional scale to evaluate possible alternative locations for the project.

Impact compensation in Brazil requires models at the scale of protected areas around the area to be damaged and which will receive resources for the mitigation of that damage [19]. Thus, three geographical scales with respect to the main decisions requested by large projects will be used to assess the performance and effectiveness of the models to guide management decisions with respect to amphibians.

The need for caution in the interpretation of niche-modeling results has been discussed in the literature [20–24]. However, understand these limitations requires a familiarity with dense and extensive scientific material, and public environmental agents do not always have time to follow the latest recommendations, since they have high workloads and little access to the specialized literature. Therefore, our goal was to evaluate the caveats in the academic literature in relation to the feasibility of applying SDMs in the decision-making process associated with environmental-impact assessment. This allowed us to make practical recommendations that will assist public agencies and consultants when planning sampling designs for impact studies and making management decisions.

## Material and Methods

### Study Area

**Fig 1:**
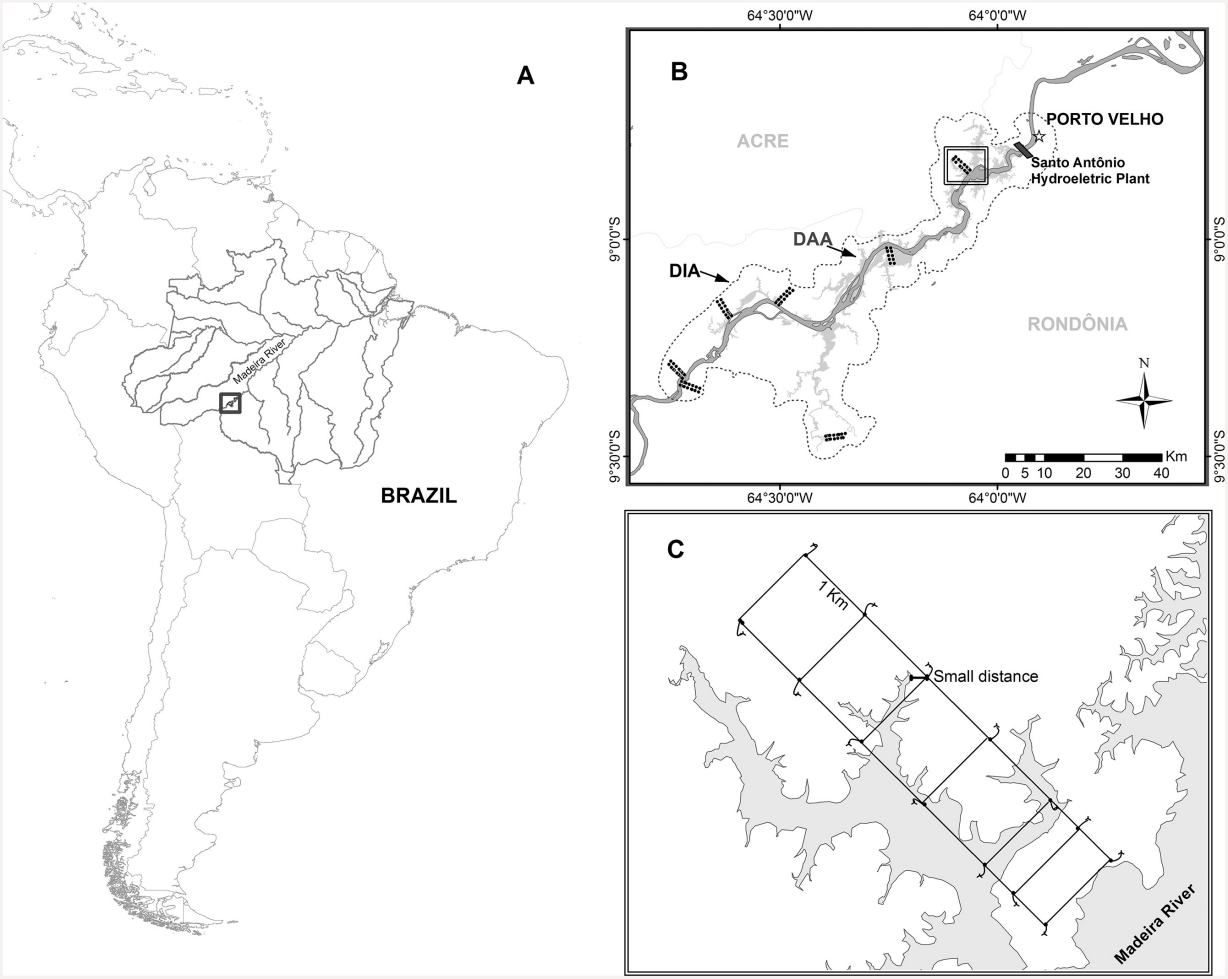
Location of the Santo Antônio hydroelectric plant, Madeira River, Rondônia, Brazil (A), and the sample area for preliminary impact studies (B). Distribution of sample modules (groups of points—B) in the direct-influence area of the project (DIA, the thin dashed line indicates the 4 km buffer around the impacted area). Dark gray area indicates the Madeira River and light gray the area below the maximum flood elevation resulting from the filling of the reservoir and represents the directly affected area—DAA. (C) Schematic drawing of a sample module perpendicular to the Madeira River in which the points indicate the start of the permanent plots. To measure the distance from threat, we considered the smallest distance between the estimated maximum limit of flooding (70.5 m altitude) and the altitude of detection of each specimen. Species detected only in the area to be submerged were considered potential targets.

The Santo Antônio hydroelectric dam is on the upper Madeira River (8°47’55”S, 63°53’55”W), about 7 km from the city of Porto Velho, in the state of Rondônia, Brazil. The dam began operation in March 2012, and flooded more than 200 km^2^ of primary rainforest. As part of the licensing process, the local herpetofauna was surveyed six times between February 2010 and November 2011, in rainy and dry seasons. Surveys were conducted in the area of direct-influence of the project (DIA) (the minimum area considered by Brazilian law), and which we use as the local scale for determining the requirements of environmental studies (Fig 1B).

The sample design was defined by a team of consultants responsible for the study, and public officials from the Brazilian Institute of Environment and Renewable Natural Resources, following legal guidelines in Brazil [17–18]. Field procedures followed the RAPELD method [25–27], and were adapted to measure the impact of the installation on elements of local biodiversity in relation to the known distributions of these elements [26]. Details of the sample design are described in the appendix (S1 Text).

In Brazil, the collection or transport of biological material; Temporary maintenance of wildlife specimens in captivity; Capture or marking of wild animals in situ for scientific or teaching purposes are analyzed and issued through the System Authorization and Information on Biodiversity (Sisbio). This system was administered by National Institute of Environment and Renewable Natural Resources—IBAMA and Chico Mendes Institute for Biodiversity Conservation—ICMBio, which are responsible for the ethical treatment of animals. Frogs were collected as part of government-mandated environmental assessment surveys, under IBAMA/SISBIO permit No 13777–2. This permit was subject to approval of all procedures for catching and collecting species and specimens.

We followed the directives of the Federal Council for Biology (CFBIO) Resolution CFBIO N° 08/12/2012, which relates to procedures for capture, containment, release and collection of vertebrates in situ and ex situ. Article 8° of that resolution states that “Collection of animal specimens, when essential to attain the objectives of the studies, research, teaching activities and general services must be undertaken with a minimum of suffering, by means of methods that rapidly induce unconsciousness and subsequent death without evidence of pain or agony using anesthetics in sufficient doses to produce painless loss of consciousness, followed by cardiovascular arrest”. According to this recomendations, all specimens collected, were sacrificed by overdose of Benzocaine 2%. Specimens were only collected in cases that genetic analysis were needed for confirmation about identification, and only two specimens were collected per locality to avoid the possibility of affecting population densities. Specimens collected were maintained individually in plastic bags with leaf litter in cool shaded locations until sacrificed within 8 hours of collection.

All species collected are of “least concern” under IUCN red list criteria.

### Target-species selection

Potential target species were defined as those only detected within or immediately close to the area expected to be flooded by the reservoir (directly affected area, hereafter DAA). To select of potential-targets, we used the smallest distance between the estimated maximum level of flooding (70.5 m) reported by the hydroelectric consortium, and the altitude of local detection of each specimen. Values equal to zero indicated points located at the edge of the flooding zone. Negative values indicate vertical distances below the maximum level of flooding, and positive values refer to locations above the maximum level of flooding. Species-occurrences were sorted by their vertical distances to the maximum level of flooding. All data are available in the data repository of the Program for Biodiversity Research (PPBio) and in the Supporting Information (S1 Table). The script for replicating the target plots is available in S1 Code.

Species widely distributed in the Amazon might be impacted locally by the installation of an enterprise, but these species might not be impacted over larger areas. Other species might be impacted at all spatial scales if they are only detected in impacted areas. To filter the potential-targets, threatened at all spatial scales, in addition to consideration of biological complementarity [28] between species occurrence in the DAA and adjacent areas (indirect-influence area of the project, hereafter DIA), we also considered complementarity of species distributions between the DAA and other regions of the Amazon. Therefore, target-species were those only detected within or in the immediate vicinity of the area expected to be flooded by the reservoir (DAA), and never recorded elsewhere in Brazil or other countries.

To evaluate the complementarity of the DAA with areas distant from those directly sampled, we sought occurrence records of potential target species in museums and online databases (SpeciesLink, GBIF, IUCN, Encyclopedia of Life) and by consulting experts. This survey was conducted between January 2012 and February 2013.

### Species-distribution models for compensation decisions

Few data are available on the geographic distributions of most species in tropical regions and what is available is usually concentrated in a few systematically sampled locations [29–30]. Thus, suitable areas for target-species occurrence may often be identified by extrapolating beyond the sampled areas, and one must use environmental suitability [22][31], because probability-of-occurrence methods require random sampling within the area of interest or within the known species distributions, to meet the assumptions of analyses [32].

To predict geographic distributions of species [33], we used the maximum entropy algorithm (MaxEnt) since it performs well when sampling is limited [30][22]. Also, MaxEnt allows the use of only presence data and can predict distributions based on either interpolation or extrapolation. Because environmental predictors are scale-dependent [34], models used different layers to reflect the ecological scenario, to maintain strong relationships with predictors (selected by jackknife), and to avoid strongly correlated variables (Pearson correlation ≤ 0.8) [22][24]. Environmental information available for training the models is described in Supporting Information (S2 Table). More precise measurements for the variables at the local scale and of interactions between species were unavailable for the target species.

We developed MaxEnt models for each frog species that we considered to be a target, within the range of the mosaic of protected areas defined by the licensing agency as eligible to receive indemnities as a result of the effects of the project. Thus, we generated measures of environmental similarity called Multivariate Environmental Similarity Surfaces (MESS) [24] with model target-species results, to analyze the extent of extrapolation and how similar environments where the species were sampled in the license studies were to those in the mosaic of protect areas. A total of 21 protected areas under municipal, state, and federal-government administrations were compensated [35].

MESS layers can be used to identify and minimize error in geographical extrapolation using species-distribution models [24] and is similar to the BIOCLIM approach. MESS, however, uses percentiles that can include negative values that will indicate sites in which at least one variable has a value outside the observed range of values in the training set, thereby indicating a novel predicted environment [24]. Knowing where novel predicted environments are will allow assessment of where models are most uninformed, and inform where it is necessary to adopt a careful interpretation of the results. We used the mask option in a single ASCII grid file that is identical in cell size and extent to those used in predictors of the target-species models [24] to generate MESS maps and evaluate environmental extrapolation in different parts of the mosaic of protect areas.

### Species-distribution models for mitigation decisions

We used two species of the family Aromobatidae that were found in the surveys during the license process and for which presence and absence data were available within and outside the region of the project [36–40]. These data are recorded in the Herpetological Collection of the National Institute for Amazonian Research, available in GBIF and SpeciesLink, which were consulted by search for scientific names between July 2012 and November 2012. Models were run in the DIA for the local spatial scale, and in the Brazilian Amazon basin where relocation decisions may be involved.

While the two species were not targets in this study, they allowed us to simulate limited sampling and thus evaluate model predictions when interpolation and extrapolation are necessary to guide environmental compensation. Species found in this family are often impacted by human interventions because of their restricted distributions, sedentary and territorial habits and short lifespan, which restrict local and regional gene flow [41].

Algorithm performance (effects of incomplete sampling) was measured using Sensitivity (the true positive rate) derived from using the minimum training presence threshold [42], in combination with the raster calculator tool in ArcGIS, and AUC (area under the curve, characteristic of the receiver) as a measure of the failure rate, which are available in MaxEnt output. Models were run at both spatial scales using all available occurrences, with 20% of the occurrences randomly assigned by the algorithm to test the models. The average of the AUC of 10 random arrangements was used for comparisons.

For maps of environmentally appropriate areas, each image prediction (raster map) was imported into ArcGIS 9.3 to reclassify suitability based on minimum training presence thresholds. This allows comparisons among the environmentally appropriate areas from each simulation along with known species distributions. Interpretations were based on a simple heuristic scheme for the BAM diagram [43–44], which summarizes the joint effects of external biotic and abiotic characteristics and dispersal on species-distribution predictions.

The two species selected for the analysis were *Allobates nidicola* [39], which was recently described with a distribution restricted to the Purus-Madeira interfluvial region, and *Allobates femoralis* (Boulenger 1883), which is widely distributed. While *A*. *femoralis* may be a species complex [45], classification follows the current understanding of the taxon [40] within current practices of SDM.

## Results

### Target-species selection

**Fig 2:**
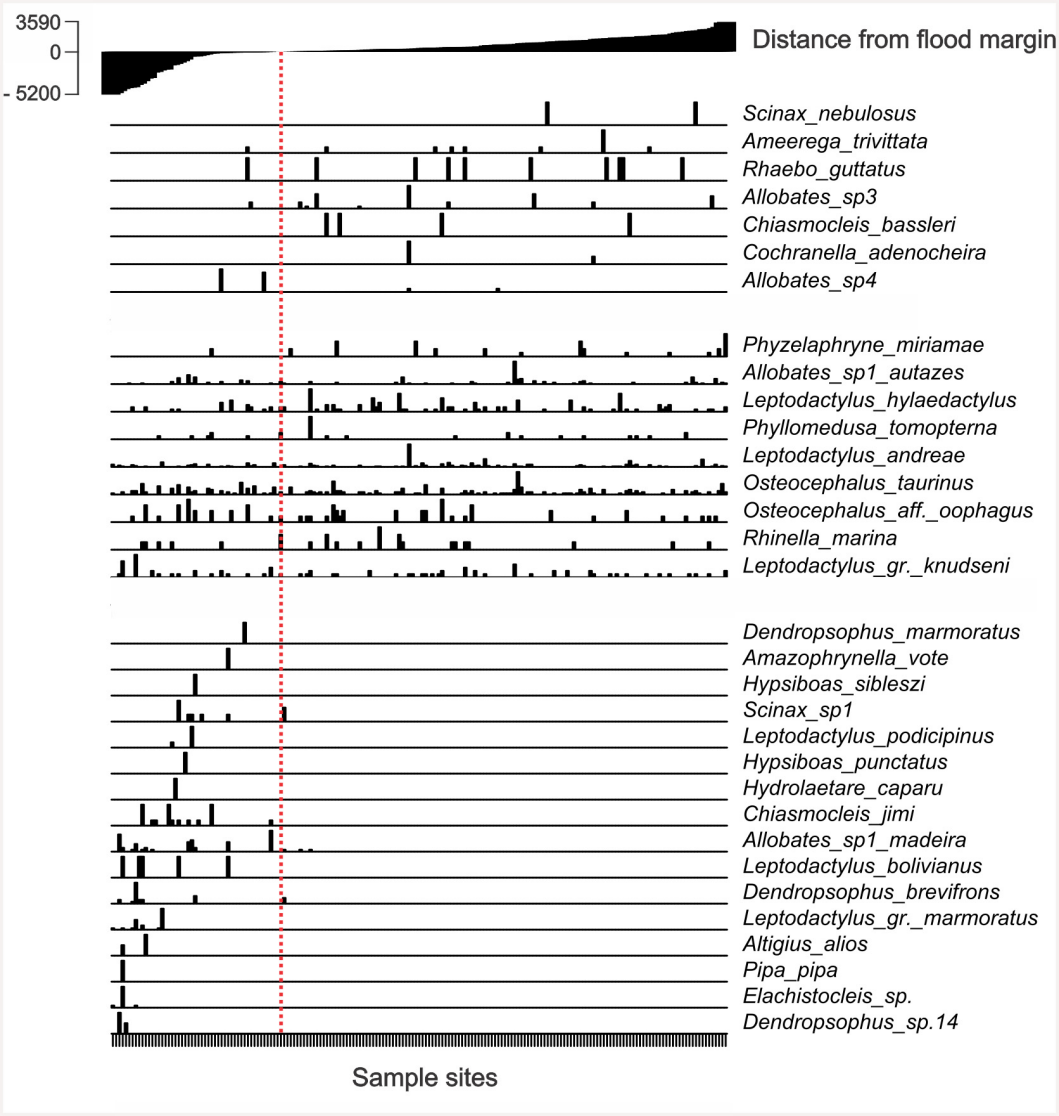
Relative abundance of species per plot in relation to the distance of the plot (m) from the expected flooded area. At the top of the chart, the level of flooding in each sample spot, with the filling of the dam.

Frog species found within the direct-influence area (DIA) may be divided into three groups with different management priorities (Fig 2). The first includes species almost exclusively found away from the area to be inundated. The second group was detected both within and away from the area to be flooded. The third group (potential target species) was only found within areas where flooding was expected (DAA) and these species were considered potentially impacted by the project.

The predicted maximum flooding by the dam was to 70.5 m above sea level. Eight potential target species had been previously recorded elsewhere in the Amazon Basin, far from the dam location and so the impact due to the dam was restricted to the scale of the project site for those species (Table 1). *Altigius alios* was previously only known outside Brazil while *Hidrolaetare caparu* had been reported from only one other location in Brazil (but also known in Bolivia, Ferrão and Lima—personal communication). Thus, these two species may be important targets for reasons of national biological heritage. Seven of the potential target species were possibly undescribed and are considered target species (scientific value) based on current legislation. Other possibly undescribed species were also detected away from the area to be flooded (e.g., *Allobates* sp3) or encountered both near and far from the area to be inundated (*Allobates* sp1 *autazes*, *Osteocephalus* aff. *oophagus*). Macro-scale distributions of these species are unknown.

**Table 1:** Species occurrences (X) and complementarity between the area directly affected by the dam (DAA) and other areas at the scale of the state (Rondônia), the Brazilian Amazon, and the entire Amazon basin (including other countries).

Target-species	Records	Status	Scale		
			State	Brazil	Amazon
*Dendropsophus marmoratus*	1	LC	X	X	X
*Leptodactylus podicipinus*	2	LC	X	X	X
*Hypsiboas punctatus*	1	LC	X	X	X
*Leptodactylus bolivianus*	5	LC	X	X	X
*Pipa pipa*	1	LC	X	X	X
*Amazonphrynella vote*	1	-	X	X	?
*Allobates* sp1 *madeira*	15	LC	?	X	X
*Hypsiboas sibleszi*	1	LC	?	X	X
*Chiasmocleis jimi*	10	DD	?	X	?
Hydrolaetare caparu*	1	DD	?	?	X*
***Altigius alios***	2	DD	?	?	X
***Elachistocleis* sp.**	3	-	?	?	?
***Dendropsophus* sp 14**	2	-	?	?	?
***Leptodactylus* gr. *marmoratus***	7	-	?	?	?
***Dendropsophus* gr. *bevifrons***	7	-	?	?	?
***Scinax* sp1**	6	-	?	?	?

Records indicate the number of independent sightings. Status indicates IUCN ratings of Least Concern (LC), Data Deficient (DD) and unclassified (-). X indicates presence, and? indicates unknown.

*Ferrão and Lima (personal communication), *Hidrolaetare caparu* was also recorded by M. Gordo in the Corumbiaria State Park; its type locality is Bolivia.

None of the potential target species was classified as threatened by the IUCN Red List or the official list of Brazilian threatened fauna species [46]. Also, none has known economic value. Fourteen potential target species (88%, Fig 2—third group) had less than 10 occurrence records for parameterization of the models at this scale. Of these species, the possibly undescribed species (*Elachistocleis* sp., *Dendropsophus* sp.14, *Leptodactylus* gr. *marmoratus*, *Dendropsophus* gr. *brevifrons*, and *Scinax* sp1) were only detected in the study site.

### Species-distribution models for compensation decisions

**Fig 3:**
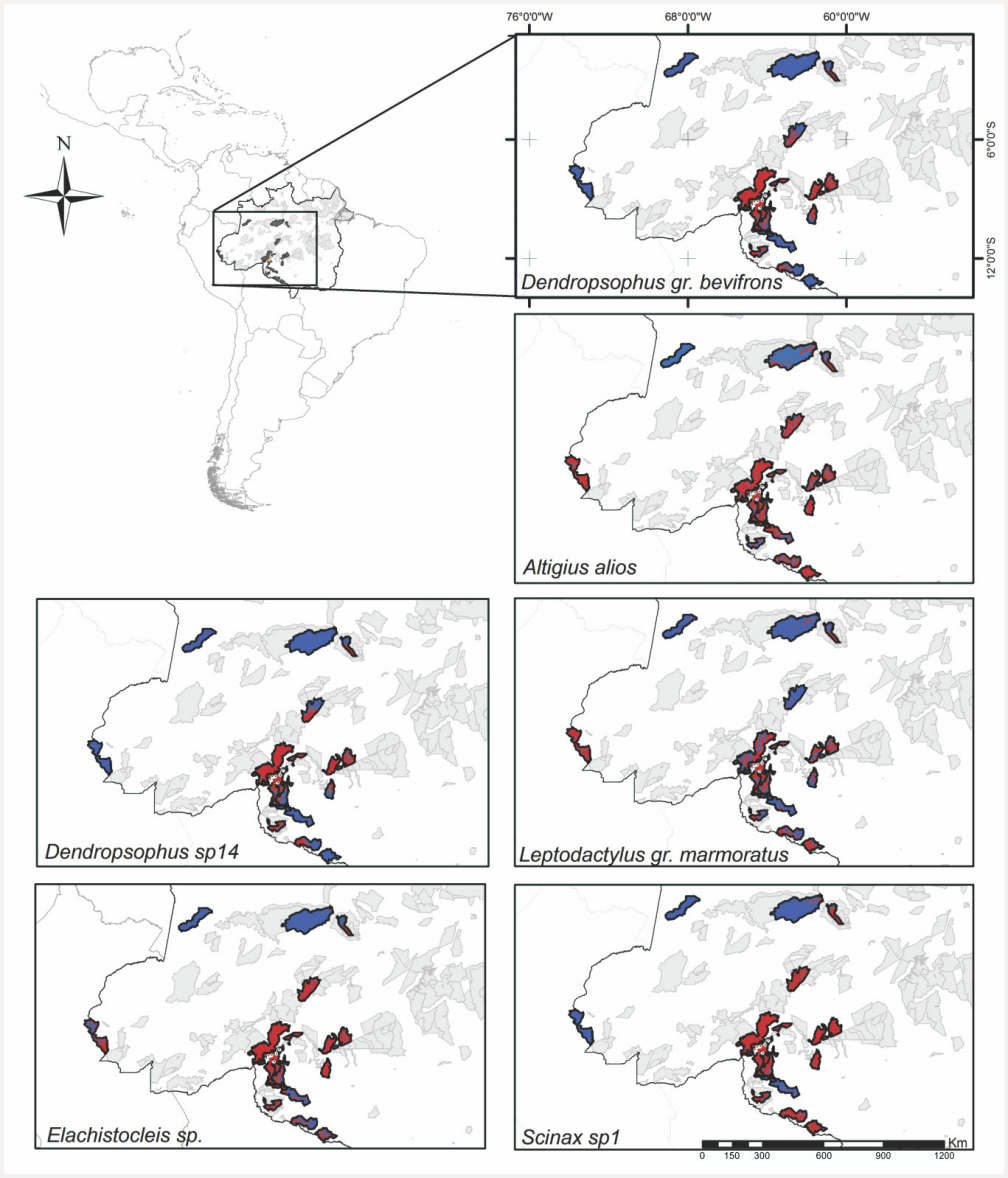
The results of multivariate environmental similarity surface (MESS) analysis for target species and the mosaic of protected areas slated to receive compensation funds. The environment in red areas was similar to that in which the study was conducted (black dots). Blue areas were dissimilar and so using models with fitted functions would not be recommended. Light gray indicates other protected areas in the region that were not included by the environmental agency for compensation.

MESS analyses for all target species found that at least one protected area among the 21 reserves was environmentally dissimilar (blue areas–Fig 3) to the sample locations. Thus, one or more variables were outside the range used to train the models, and therefore the locations have novel environments. In novel environments the results of species distribution models are unreliable.

### Species-distribution models for mitigation decisions

**Fig 4:**
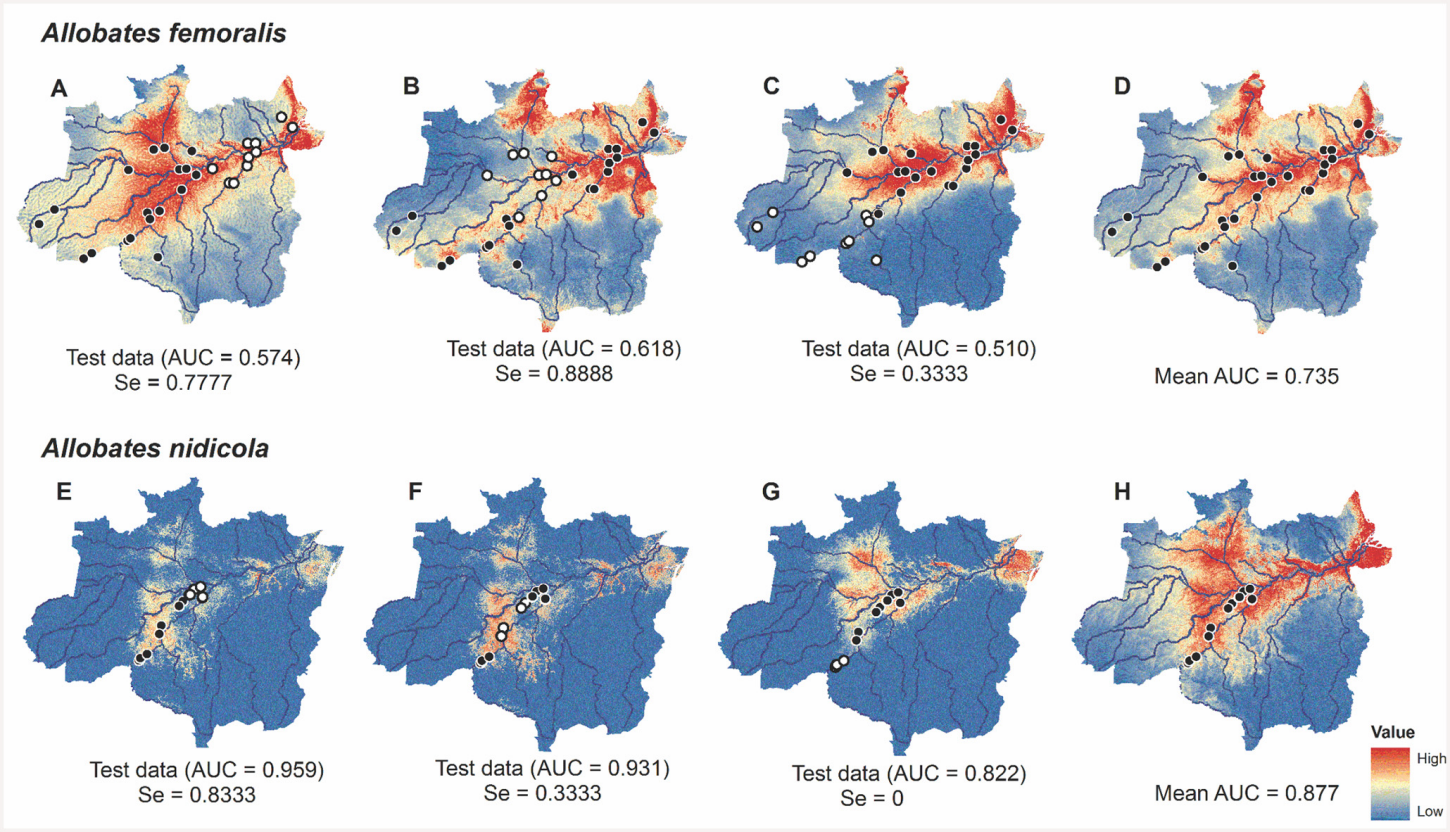
Predictions of environmentally suitable areas for the occurrence of *Allobates femoralis* and *Allobates nidicola* at the scale of the Brazilian Amazon Basin. Occurrences for each species were separated into 3 groups based on distance for evaluating predictions using model interpolation and extrapolation when sampling was incomplete. Training records for the simulation models are black and validation points are white. Models D and H included all available occurrences of which 20% were randomly assigned by the algorithm to test the models.

**Fig 5:**
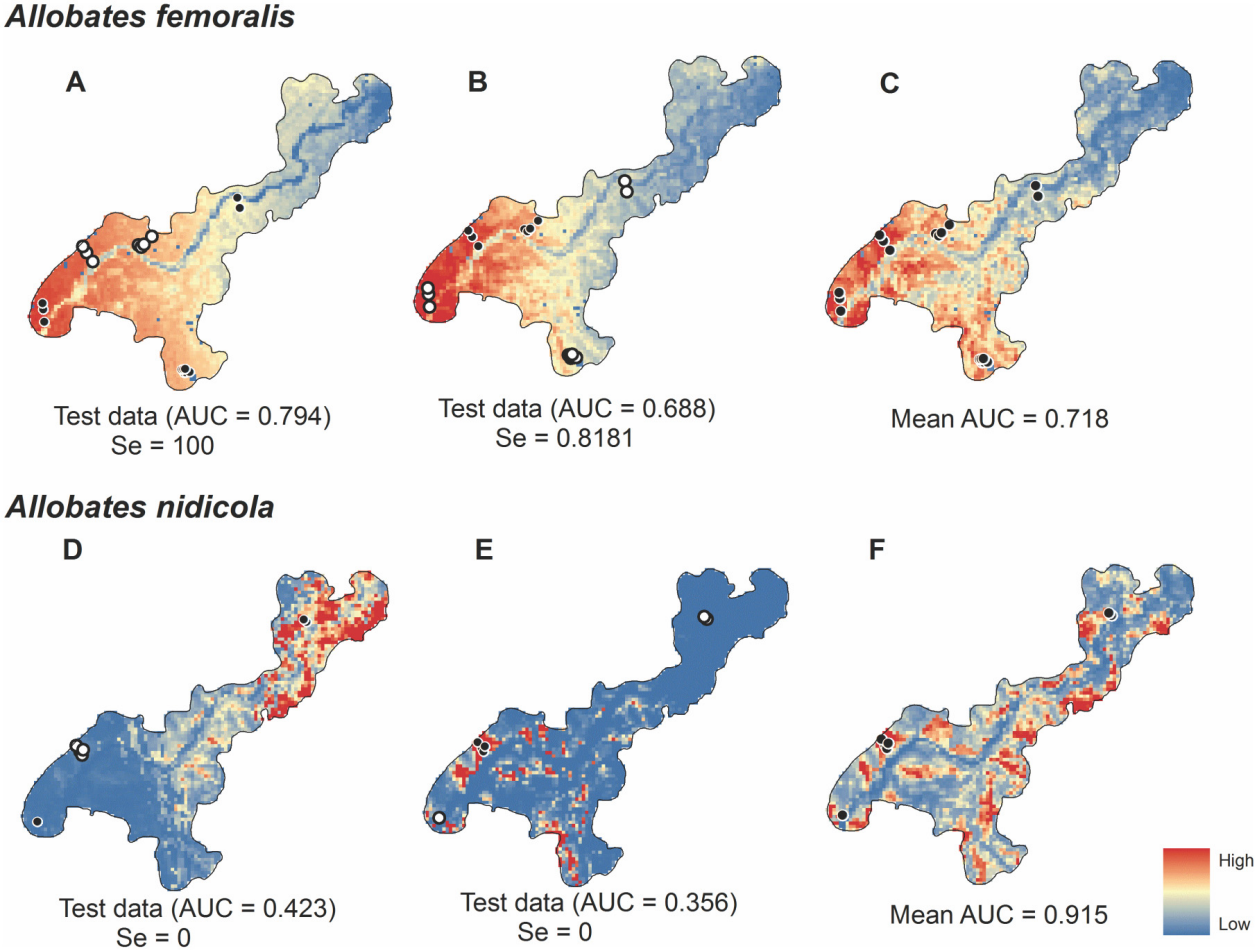
Predictions of environmentally suitable areas for the occurrence of *Allobates femoralis* and *A*. *nidicola* at the local scale (DIA). Two groups simulated extrapolated and interpolated predictions. Points in black were used for training and white dots indicate test records. Models C and F included all available occurrences, 20% of which were randomly assigned by the algorithm to test the models.

Models based on known species distributions at both spatial scales generated variable predictions when used with different portions of the data for model training. This result was evident in AUC and sensitivity testing (Fig 4). Lower AUC and sensitivity values resulted when test records were predicted by geographical extrapolation of training records (Figs 4C and 4G and 5B). Errors of omission and commission were found for the Amazon-wide models for *A*. *nidicola* because the species was predicted to be widespread in many parts of the basin though it is known to be restricted to the Purus-Madeira interfluve. Greater environmental suitability was predicted along the lower reaches of the Amazon River (where it does not occur) than in the region where it does occur.

## Discussion

### Target-species selection and species-distribution models for impact assessments

Our results show that the species most in need of conservation in tropical regions are those for which Species Distribution Modeling is less likely to be accurate [25]. Indirect negative impacts can affect key species far from the flooded areas. However, due to the short time during which measures can be proposed and the few studies using comparable sampling designs, damage is unlikely to be determined until much later in the impact evaluation. Given the rapid expansion of infrastructure projects in forested areas, data produced in the impact assessments must be integrated to anticipate these indirect impacts and to inform planning for future studies across the Amazon basin and mitigation decisions at larger geographic scales. However, few Latin-American countries require evaluation of cumulative impacts [6] and there are many gaps in methods, guidelines, and regulation definitions governing assessment of cumulative impacts at large scales.

### Species-distribution models for compensation decisions

Compensation policies for environmental impacts are legislated in most of the countries with Amazonian territories [6]. In Brazil, compensation funds are considered to be important sources of finance for reserve administration. The National System of Protected Areas [47] states that, in cases of environmental licensing of impacting ventures and based on the impact report, the implementation or maintenance of protected areas must be supported by the entity causing the impact.

Although environmental compensation is assessed in accordance with the impact caused by the project, the global analysis illustrates the magnitude of the geographic scale usually considered in compensation decisions in the Brazilian Amazon, and the extent of environmental extrapolation necessary to make decisions. Thus, SDM as a tool to assist in determining compensation [4], may often be incomplete for determining the environmental suitability of distribution areas of target species as a criterion for the definition of protected areas [4]. Thus, using MDE for predicting environmental suitability is likely to be uncertain with undesirable consequences for conservation policy planning, especially when models predict areas having environmental characteristics unlike those of the area used to calibrate the model [23][48].

Extrapolations are susceptible to errors of omission [49] because the data used for model parameterization cannot represent all conditions in the extrapolated region. MESS analysis has been used to evaluate similarity or novelty of environments to indicate where models are best informed and to guide decisions based on these predictions and to assist in model interpretation [24]. Additionally, MESS can be used to identify and reject models with fitted functions that extrapolate in ways that are biologically implausible.

An important restriction on the use of these tools is in regard to space disagreement between sampled areas, which are typically defined by licensing agents as the region adjacent to the affected area (Fig 1B), and those areas in which decisions concerning compensation must be made, which are typically outside that area. For example, the goal of compensation actions is to permanently or temporarily compensate for the use of natural resources that become unavailable. In a scenario that selects regions for compensation, models must extrapolate beyond the data that are available for calibration and, based on those extrapolations, predict environmentally appropriate areas for the target species. As seen in the models described herein, at least one protected area was not representative of the data used for training in all target models. Therefore, the SDM tools for guiding management decisions may often produce uncertain results and may not be better than other approaches, such as the use of expert opinions.

Distribution-modeling tools may be useful for improving sampling requirements in environmental-impact studies [4]. Nonetheless, the scarcity of occurrence records for target species remains a major limitation. Models are clearly influenced by the number of records used for predictions [11–12][50–52] and using such methods must be restricted to species with a sufficient number of occurrence records at all scales. In the case of Santo Antonio, model use was restricted to species that are not considered immediately impacted by the project. Thus, in Brazil, models can only assist in other stages of the licensing process (e.g., post-impact monitoring) or to request clarification or supplementation to the analyses of the studies [53]. That is so because, in Brazil, sample design and sample sites in environmental studies are predefined in the Terms of Reference, and based on secondary biodiversity information for the area that will be impacted. As secondary information is not available for the vast majority of Amazonian regions, this precludes the use of these tools to guide the installation of sampling units in the initial stage of the study or to make predictions at wider scales.

### Species-distribution models for mitigation decisions

Simulations with data on *Allobates nidicola* and *Allobates femoralis* showed that, at the local and Amazon-wide spatial scales, models for which geographic predictions were extrapolated beyond the training area had poor predictive performance. It is well-known that projections of models through time and space can have varying results [23][54–56], and are not recommended for the guidance of policy decisions without prior validation.

Problems posed by extrapolation are likely to be even greater in the Amazon because historical factors affecting distributions, which are difficult to quantify, are likely to be more important than the available environmental data [57–58]. Studies show that biogeographical processes influence the distribution patterns of organisms in Amazonia [37][59–62], and many of these historical processes remain unknown. Disagreement continues about the most important historical events that occurred in the region [63–64]. Such historical and geographical processes may be independent of the current environmental conditions, even with the inclusion of information regarding past climates and paleolandscapes. Directed studies of target species are necessary to evaluate other possible dispersal barriers.

Critical points for the formulation of robust models can be seen in the BAM diagram [15] [43–44]. When estimates of the area of occurrence are required for compensation or project relocation, studies must ascertain favorable conditions for the species (environmental predictors simulating the fundamental ecological niche) and factors that limit species distributions (biotic and historical factors that restrict geographic dispersion). Otherwise, errors of commission will be inevitable [15][65], as seen for *A*. *nidicola* at the large spatial scale. Model predictions indicated greater environmental suitability for the species near the mouth of the Amazon River than in the region where it occurs (Purus-Madeira interfluvial region).

Despite limitations, species-distribution models may be useful to assist in conservation by contributing to strategic decisions about environmental impacts in the tropics [29]. For this usefulness to be realized, environmental agencies must develop strategies that reduce uncertainty in predictions of species distribution, along with improving understanding of the biodiversity of each region. Systematic sampling within the area of interest is the most efficient way to developed unbiased models [56].

Few countries in Latin America require evaluation of cumulative impacts [6] over wide areas as part of EIA scoping. In addition to gaps in records of species occurrences, the lack of locally and regionally integrated methods, guidelines and regulations prevent effective cumulative-impact assessment.

An alternative for overcoming the challenges we describe here may be to simply expand the sample area defined by the licensing agents to include a larger scale near the affected area. The use of permanent plots with multiple surveys over a wider area will provide data on the detection probabilities to better understand the probability of false absences. Sampling larger areas will also allow interpolation (rather than extrapolation) based on data collected and thereby reduce inaccurate predictions. Refined, data-informed, environmental variables may also be collected in the field to produce better local-scale models.

The quality of the data determines the quality of estimation of species richness at multiple scales and is likely to be the major challenge for ecogeographical studies [66]. Alternative conservation strategies should be to invest in programs of long-term ecological research that are integrated with surveys to monitor regional biodiversity and which should be undertaken prior to the short-term studies that are associated with licensing individual sites [26][34].

## Conclusions

Poorly understood species distributions pose a risk to biodiversity when potentially damaging enterprises are licensed in the Amazon, because the required studies are spatially limited and many new species may often be found in surveys. Because of this limitation, the use of modeling tools to overcome such deficits and to guide requirements in impact assessments remains challenging, because of the large scales involved in management decisions, and the lack of secondary data for calibration of the models.

SDM often may be useful to guide and inform some aspects of the licensing process in the Amazon, but the size and placement of the sampling areas defined by the licensing agents must be established based on modeling considerations, rather than simply close to the impact area of the project. Also, programs that integrate long-term ecological research with surveys to monitor the biodiversity in the region will be important.

Because of the many infrastructure projects that are planned for the Amazon, it is important that the information required by the environmental licensing procedures consider cumulative impacts, and that they be based on methods that allow evaluation of biological complementarity over regions in the basin and enable the use of more realistic models with robust regional information in the future.

## Supporting Information

S1 Code R Script code for generic graph. The amphibian occurrences and abundances ordered in relation to the flood margin predicted of the dam.
(DOC)

S1 Table Location, elevation and the number of individuals of species detected in amphibian surveys during the environmental-impact studies for the Santo Antonio hydroelectric dam. (XLS)

S2 Table Environmental information and their sources used to calibrate the prior models. The layers were selected according to each species and scale.
(XLS)

S1 Text Sample design of the Environmental Impact Study adopted in the Santo Antônio Hydroelectric Dam licensing process. Six surveys of the local herpetofauna were conducted.
(DOC)
