# Supplementary material for: Limitations to the Use of Species-Distribution Models for Environmental-Impact Assessments in the Amazon
Source: PLoS One. 2016 Jan 19;11(1):e0146543. doi: 10.1371/journal.pone.0146543 (PMC4718640; doi:10.1371/journal.pone.0146543)
Supplement: S1 Code — The amphibian occurrences and abundances ordered in relation to the flood margin predicted of the dam. (DOC) [file pone.0146543.s001.doc]

**S1 Code. R Script code for generic graph.** The amphibian occurrences and abundances ordered in relation to the flood margin predicted of the dam.

# Function to Generic Graphic adapted

### Created by Victor Lemes Landeiro #####

#### Species detected in DIA

especies<-read.table("ADI.txt", header=T)

spp<-especies[,2:17]

inund<-especies[,1] #checar 1:2

spp.medias<-colSums(spp*inund)/colSums(spp) #rever

spp.sort<-sort(spp.medias)

spp.ord1<-spp[order(inund), ]

spp.ord<-spp.ord1[,order(spp.medias)]

####### Species detected in DAA

especies<-read.table("AII.txt", header=T)

spp<-especies[,2:9]

inund<-especies[,1] #checar 1:2

spp.medias<-colSums(spp*inund)/colSums(spp) #rever

spp.sort<-sort(spp.medias)

spp.ord1<-spp[order(inund), ]

spp.ord<-spp.ord1[,order(spp.medias)]

##### Species generalist

especies<-read.table("espalhadas.txt", header=T)

spp<-especies[,2:11]

inund<-especies[,1] #checar 1:2

spp.medias<-colSums(spp*inund)/colSums(spp) #rever

spp.sort<-sort(spp.medias)

spp.ord1<-spp[order(inund), ]

spp.ord<-spp.ord1[,order(spp.medias)]

### Graph

generico<-function(tabela,gradiente,at,grad,eixoY,eixoX,excluirspp){

tabela<-as.matrix(tabela)

gradiente<-as.matrix(gradiente)

media.pond<-colSums(tabela*gradiente[,1])/colSums(tabela)

sub.orden<-tabela[order(gradiente[,1],decreasing=F),] # Ordenar parcelas de acordo com o gradiente

sub.orde<-sub.orden[,order(media.pond,decreasing=T)] # colocar espécies ordenadas pela média ponderada

dados.pa<-matrix(0,nrow(tabela),ncol(tabela))

dados.pa[tabela>0]<-1

ordenado<-sub.orde[,which(colSums(dados.pa)>excluirspp)] ## para deletar possíveis colunas vazias (espécie que não ocorreu)

par(mfrow=c(ncol(ordenado)+1,1),mar=c(0,4,0.2,10),oma=c(3,1,1,6))

layout(matrix(1:(ncol(ordenado)+1)),heights=c(3,rep(1,ncol(ordenado))))

plot(sort(gradiente[,1]),axes=F,ylab="",mfg=c(21,1),lwd=10,las=2,lend="butt",frame.plot=F,xaxt="n",type="h",col="black",ylim=c(min(gradiente),max(gradiente)))

axis(side=2,at=c(0,max(gradiente)),las=2)

mtext(grad,4,outer=T,font=2,line=-10,padj=-18.5,las=2)

for(i in 1:ncol(ordenado)){

if(i == ncol(ordenado)){

barplot(ordenado[,i],bty="l",axisnames=T,col.axis="white",axis.lty = 1,names.arg = 1:nrow(ordenado),cex.names=000.1,axes=FALSE,col="black")

#axis(side=1,at=c(1:nrow(ordenado)),las=2)

}

else{barplot(ordenado[,i],bty="l",axisnames=F,axes=FALSE,col="black")}

#axis(side=1,at=max(ordenado[,i]),las=2)

mtext(colnames(ordenado)[i],1,line=-1.0,adj=0,at=at,cex=.7,font=3)

}

mtext(eixoX,2,outer=T,font=2,line=1.2)

mtext(eixoY,2,font=2,outer=T,line=-2)

}

### Graphical editing

par(mfrow=c(2,2))

generico(spp.ord,inund,230," ", " "," ",0.3)
