# Supplementary material for: Limitations to the Use of Species-Distribution Models for Environmental-Impact Assessments in the Amazon
Source: PLoS One. 2016 Jan 19;11(1):e0146543. doi: 10.1371/journal.pone.0146543 (PMC4718640; doi:10.1371/journal.pone.0146543)
Supplement: S1 Text — Six surveys of the local herpetofauna were conducted. (DOC) [file pone.0146543.s004.doc]

**S1 Text. Sample design of the Environmental Impact Study adopted in the Santo Antônio Hydroelectric Dam licensing process.** Six surveys of the local herpetofauna were conducted.

Sampling was undertaken in seven modules, each consisting of two 5km trails perpendicular to the original course of the river, systematically distributed within the direct-influence area (DIA). The arrangement of the modules perpendicular to the river was used to assess the impact of the flooding gradient on the species.

Along each trail, seven 250-m long permanent plots were installed at one-km intervals beginning at the riverbank. Extra plots were installed at zero and 1000 m to better evaluate the riparian areas. The plots followed the altitudinal contours to minimize the internal variation in the distance to the water table and soil characteristics. Frogs were sampled using an active search technique by two observers that were limited by time, pitfall traps installed in permanent plots, and random encounters along the access trails.

Six surveys of the herpetofauna were conducted between February 2010 and November 2011, which included surveys in high- and low-rainfall seasons (February-April 2010, August 2010, November 2010, January 2011, May-June 2011, October 2011).

Active search limited by time was used in each sampling plot. This methodology consisted of visual and auditory detection along of the center line of each plot, which was 250 meters long. The number of each species encountered was recorded. The sampling time was controlled for each plot, so as to have similar effort in different plots and modules. At each sampling occasion, plots were surveyed by two people (one expert and a field assistant) during one hour. All plots and modules were sampled in each survey campaign.

Sampling was undertaken in three periods: daytime (09:00-16:00h), twilight (16:30-18:30h) and night (19:00-23:00h), so it was possible to detect and quantify species differing in activity period. The sampling period varied according to the season most appropriate for the sampling different groups. At the end of the rainy season (campaigns 1 and 4) plots were sampled only during twilight and night periods, when there was a greater number of records of frogs and nocturnal snakes. During the dry season (campaigns 2 and 5) surveying was carried out during the day shift when lizards were active and few frogs were vocalizing. At the beginning of the rainy season (campaigns 3 and 6), sampling was carried out in the three periods.

When actively searching at night, each pair of observers carried a portable digital recorder and recorded the vocalizations of individuals during sampling. The records were used to confirm the identification of species by AP Lima, an expert who was present during all campaigns. The search time was approximately one hour per plot (14 hours per module).

Specimens collected were maintained individually in plastic bags with leaf litter in cool shaded locations until sacrificed within 8 hours of collection, and they were deposited in the Herpetofauna collection of the National Institute for Amazonian Research. All species collected are “least concern” under IUCN red list criteria.

Frogs were collected as part of the government-mandated environmental assessment surveys, under IBAMA/SISBIO permit No 13777-2. This permit was subject to approval of all procedures for catching and collecting species and specimens. We followed the directives of the Federal Council for Biology (CFBIO) Resolution CFBIO N° 08/12/2012, which relates to procedures for capture, containment, release and collection of vertebrates in situ and ex situ. Following those recommendations, all specimens collected, were sacrificed by overdose of Benzocaine 2%.
